# The Need of Oral Prophylaxis and Some Results Obtained by It

**Published:** 1905-10-15

**Authors:** Edward B. Spalding


### THE NEED OF ORAL PROPHYLAXIS AND SOME RESULTS OBTAINED BY IT.

EDWARD B. SPALDING, D.D.S.

Read before the Michigan State Dental Association, July 11th, 1905.

Prevention is the spirit of the times in business and in the professions.

Large corporations employ the best minds in the legal profession whose duty it is to guard against and prevent conditions which may result in loss of life, time and capital.

The medical profession is devoting a very large portion of its thought towards the prevention of disease.

Only recently an article appeared in a medical journal, in which was advocated the monthly examination of apparently healthy persons, thereby being able to detect disease, in its incipient stage, remove the cause and allow the system to return to its normal condition of resistance.

Some of the larger cities require the frequent medical examination of all children attending the public schools, and no child is permitted to enter a school unless he holds a certificate from a medical examiner.

A similar examination of the mouth and teeth of each pupil by a competent dentist would save many from suffering, and instruction in the care of the mouth would prevent in the schools the spread of many diseases, germs of which breed in an unclean mouth.

### CONDITION OF THE HUMAN MOUTH.

There exist pathological conditions in the human mouth, a correction of which would mean much toward lessening the necessity for mechanical substitution of partially or wholly destroyed dental organisms. Also the general health would be so materially advanced that it is surprising the dental profession is only beginning to realize the possibility of correction and prevention of these conditions.

### DENTISTS HAVE NOT RECOGNIZED AND REMOVED THE CAUSES OF MOUTH DISEASES.

Formerly when a patient presented himself with teeth seemingly melting away with the white, rapid decay, he was readily told that he had very soft teeth, and that he could not have gold or other so-called permanent fillings inserted, for such fillings would not last in his mouth. Rarely has the dentist been careful to remove all accumulations, seen and unseen, which were on and about the teeth in such a mouth before beginning his prided work of filling. The condition of lactic acid fermentation was allowed to remain uninterrupted until all the work of filling had been accomplished, and then the disagreeable operation of "cleaning the teeth" may have been done in a very perfunctory manner. The dentist may have prescribed an alkaline mouth wash, and it may have been productive of considerable temporary good by neutralizing a portion of the acids present. But it did not remove the media upon which the germs of fermentation were growing, neither did it destroy the germs themselves.

So, too, the patient suffering from pyorrhea was told his case was hopeless, that any treatment would be merely palliative, and that it was only a matter of time before he must loose his teeth and substitute an artificial denture.

Let us think for a moment how the teeth were intended to be used, what is nature's environment for them, and then, how they are used and their present environment.

### NATURE'S PLAN.

Man in his primitive stage was strong in every organ and part of his body. All tissues received vigorous use, and as proper use strengthens, so they were maintained in health.

His foods, both flesh and vegetable, at first raw, but at later periods cooked, were always of a coarse, fibrous nature, nearly devoid of taste unless thoroughly masticated and required vigorous use of his teeth to reduce them for delutition. During this process, that of mastication, it was necessary for the peridental membrane to withstand many pounds of intermittent pressure from the teeth, the gums much massaging and the enamel much friction. It was their normal exercise which produced a healthy circulation and resistance in the parts.

From the nature of his foods there was little of a sticky tenacious substance to ahere to his teeth, and were it to do so, it could not remain longer than until his next meal, for then came the broom like sweeping over his teeth of the coarse foods again completely removing any particles left from his previous meal. True, he undoubtedly had several kinds of bacteria in his mouth, but as the culture media was scarce, they could not multiply and form acids and poisons rapidly enough to do him and his mouth tissues injury. In other words, he had perfect health and perfect teeth given him, he gave the latter exercise of a proper kind and did not surround them with irritants and those substances which tend to produce a pathological condition.

THE PLAN OF CIVILIZED MAN.

Now let us contrast the case of the person living in the present state of civilization.

In a very large precentage of cases, the person to-day has imperfect occlusion of his teeth, consequently, to begin with, he cannot give his teeth their proper exercise, were his foods suitable, and his inclination to do so.

The most of his food is so finely prepared, thoroughly cooked, and highly seasoned, that when taken into his mouth there is little desire to use the teeth on it, and much of it is so rich that if it were much masticated, it would immediately become sickish to the taste.

The habit of hurrying in every thing he does to-day, man applies to his eating also.

The consistency of the foods is mostly of a sticky, doughy, tenacious nature, adhering readily to the teeth, and offering no resistance to them during mastication. The cooked starches combine readily with the other accumulations and the secretions of the mouth to make a most suitable media for the growth of bacteria. Consequently there is constantly more or less of a coating of this material upon many if not all surfaces of the teeth of every one to-day. Were there sufficient friction of mastication these accumulations would be worn off frequently, but there are many locations on and about the teeth which retain their coatings months at a time. Of course, some portions of the accumulations are worn away and pass into the stomach with the food, but they are continually replaced by other and fresh media suitable for bacterial growth.

The lactic acid continually formed by the fermentation of the starches finally softens the enamel of the teeth and caries begins. The gum also becomes inflamed by the same cause and from the toxins formed in the putrefaction of the nitrogenous matter. The teeth and gums in this condition being sensitive, mastication is now entirely avoided in that locality where there was at least partial use of the teeth before. After use ceases in any locallity, the coatings on the teeth increase.

Now we started with a condition which would not permit of normal, healthful use of the teeth, caused first by malocclusion; second, by the consistence and richness of the foods; and third, the habit of rapid eating, and we end with complete disuse of the teeth in a certain locality, due to sensitiveness from caries and inflammation of the gingivae, and with lowered resistance in all of the tissues of the mouth.

NUTRIENT MEDIA FOR MICRO-ORGANISMS IN THE MOUTH.

Now let us see what this sticky, slimy material consists of which is found in a more or less degree on the teeth of every one to-day, and is the soil for the growth of many microorganisms. W. D. Miller says: "The organic and inorganic substances found in the mouth, which may serve as nutriment for micro-organisms are the following:

1. Normal saliva.
2. Buccal Mucus.
3. Dead epithelium.
4. Dental tissue softened by acids.
5. Exposed pulps.
6. Exudations of the gums, conditioned by the irritation of tartar, etc.
7. Accumulation of particles of food."

The last named component in the nutrient media, particles of food, is, of course, the most abundant and subject to the most variation.

These seven substances in combination seem to form a most ideal pabulum in the mouth for the growth of bacteria and many micro-organisms grow and thrive in the mouth which no one has as yet been able to cultivate artificially.

MANY VARIETIES OF BACTERIA IN EVERY MOUTH.

Miller has isolated over a hundred varieties of bacteria from the mouth. There are found constantly in every human mouth at least six different varieties, and in every mouth there are many others which may be regarded as more or less transient.

The soft deposits are continually loaded with these bacteria, which vary in quantity and kind with the opportunity of entrance to the mouth, and with the abundance and quality of the deposit, for many of the mouth bacteria are very sensitive to a change of media.

LACTIC ACID AND POISONS PRODUCED BY THE BACTERIA.

A large percentage of these bacteria are capable of producing fermentation and lactic acid in the presence of carbohydrates, and many others produce products during their growth on the albuminous substances the same as those found generally in the putrefaction of nitrogenous matter.

### DECAY OF THE TEETH AND INFLAMMATION OF THE SOFT TISSUES CAUSED BY LACTIC ACID AND OTHER CHEMICAL IRRITANTS.

Now we know that the products of fermentation and putrefaction produce intense irritation in the soft tissues, and that lactic acid is accountable for the solution of enamel in the commencement of caries at least. This we learn from the researches of Miller, Black, Williams and others, as also that the bacteria produce a gelatinous substance which adheres to the surfaces of the teeth, and that the acids there produced are held in close contact with the enamel. These bacterial coatings or plaques are always closely associated with caries.

These bacterial films attached to all teeth, but especially to those surfaces not worn by mastication, and unclean teeth. Miller says: "We may put it down as an axiom that films will be found wherever the surface of a tooth is not kept free from deposits of mucus, epithelium, food, etc." He further says: "It would be very difficult to find a tooth which did not show bacteria adhering to the surface at some point or other."

To further emphasize the fact that the coatings, deposits and films are injurious to the teeth let me quote from an article by J. Leon Williams, which appeared in the *Dental Cosmos* for April 1899: "The fluctuations in the decay of the teeth which we have so long observed are due not to change in tooth structure, an hypothesis which could never be held for a moment by those who understand how slow the changes in dentine are and how impossible any true physiological change in enamel must be, but no changing conditions of the environments of those micro-organisms, which constitute the sole exciting cause of the dental caries."

Undoubtedly there are other factors in the progress of caries, such as the natural resistance in the tissue themselves but, nevertheless the reduction and removal of the acid-forming material is accompanied with lessening, if not complete cessation of caries. The factor of the individual's care of his own mouth is not taken into consideration, for while he may do much toward keeping it in a state of health, when once it is made so by treatment, he cannot bring about a healthy condition himself.

### HARD AND SOFT DEPOSITS—THEIR ACTIVE INFLUENCE.

The dentist has long looked upon the calcareous deposits as the chief, if not the sole cause of irritation to the gingivae and pericementum. This, I think, is largely a mistake, for while these deposits are very irritating it is the fermenting and putrefying soft accumulations which are the most vicious in their action on the soft tissues. The hard deposits make a roughened surface which serves the better for the attachment of the softer and more irritating ones.

The removal of the tartar from the teeth is attended with some lessening of the irritation in the gums and pericementum, but the most marked change is noticed when the soft, smeary coatings and bacterial films are properly removed, then the inflammation, rapidly subsides.

### FERMENTATIVE AND PUTREFACTIVE PRODUCTS IN THE MOUTH AND THEIR INFLUENCE, LOCAL AND SYSTEMIC.

The whole mucous membrane of the mouth and pharynx is lowered in its resistance to disease by the constant presence of fermentative and putrefactive processes and products.

It has been proven that there are fewer bacteria in the mouth after a meal than before, and the swallowing of mouth accumulations containing micro-organisms and their products cause much disturbance not only of the digestive tract but of the whole system. I quote as authority for this statement Miller, Micro-Organisms of the Human Mouth, and Dental Pathology, by Burchard, revised by Inglis. In my own practice the past year a patient under treatment for pyorrhea alveolaris has been very much relieved of intestinal trouble with which he had been suffering for at least two years.

The appetite is largely dependent upon the conditions of the mouth. There are cases on record where there has been complete loss of appetite, due to fermentative and putrefactive processes in the mouth.

I think we will grant that it is necessary to reduce the fermentation and putrefaction continually taking place in the oral cavity, but how shall it be done.

### ANTISEPTICS OF LITTLE OR NO AVAIL IN THE MOUTH.

The use of antiseptics for this purpose has been productive of but little good, because the strength of solutions permissible in the mouth is not sufficient to destroy many of the bacteria, unless allowed to remain several minutes, and even then the solutions will not penetrate and remove the coatings. *The only effectual way of reducing the number of micro-organisms in the mouth is by removing the media upon which they grow.*

### DR. RIGGS AND HIS METHODS.

While Dr. Riggs believed not only in the successful treatment of the disease which bears his name, but, also, that the mouth could be so carefully treated that decay of the teeth be entirely prevented, his methods and ideas seem not to have been grasped by the profession. Therefore the successful results which he claimed have seldom been produced by them.

### DR. SMITH'S METHODS SUCCESSFUL.

It seems to have been left to Dr. D. D. Smith, of Philadelphia, to evolve a systematic method of detecting and thoroughly removing all accumulations and bacterial coatings from the teeth, stimulating the dental organs and maintaining all the mouth tissues in a state of health. He relies not upon medicaments, but upon a method which is most like nature's own way of freeing the teeth of injurious accumulations. It is that of friction from wood charged with an abrasive material sufficient to cut through the tenacious coatings covering the teeth. This treatment is applied by hand, for one must rely upon the sense of touch to determine the condition of each and every tooth surface. The most injurious materials upon the teeth are invisible, so a cultivated sense of touch is necessary to distinguish and remove them.

### ORAL PROPHYLAXIS—WHAT IS IT.

Dr. Smith calls this treatment *prophylaxis* treatment as distinguished from *prophylactic* treatment which embraces the use of therapeutic remedies.

To give his own definition: "Prophylaxis is an art or surgical treatment involving manipulative effort, as distinguished from the administration of a systemic medicament or a therapeutic remedy. Hence oral prophylaxis implies surgical instrumentation, or treatment of the mouth and teeth in contra-distinction to a germicide, a wash, or any form of medication for the prevention of disease in the oral cavity."

He further says: "The treatment consists of enforced radical and frequent change of environment for all teeth and all mouth conditions, and the maintenance of perfect sanitation for the oral cavity."

To be more specific, the treatment is, first, the most careful and thorough removal of all calcic, solid and semi-solid deposits from all exposed surfaces of the teeth and from under the free margins of the gum, using instruments principally of a file nature.

Second, with flattened, spatula-shaped orange wood points in hand porte polishers, charged with finely powdered pumice stone, every exposed surface of each tooth is polished until the slippery, slimy feeling disappears, and a smooth surface of enamel is obtained. This vigorous rubbing of the teeth and massaging of the gums gives a very perceptible stimulation to the parts, and after a few treatme ts, is very pleasant to the patient.

RESULTS NOT OBTAINED WITHOUT FREQUENCY OF TREATMENT.

This radical changing of the environment of the teeth is persisted in with frequency, varying with the needs of the case, from every third day to once a month. I find that the average untreated mouth is brought into a comparatively healthy condition with a treatment once a week for from three to five times, and then once a month.

To maintain the mouth in good condition, a month seems to be as long as it should go without a treatment.

*One, two or three treatments does not constitute prophylaxis. It is the constant watching, guarding and maintaining the mouth in a condition of health which constitutes oral prophylaxis.*

INSTRUCTION OF PATIENT AND ENLISTMENT OF HIS CO-OPERATION.

It includes also the instruction of the patient, insisting that he co-operate with the dentist by the use of the pick, the floss, and the tooth brush and powder. Without his help, the operator cannot attain the most satisfactory results. The patient rarely, if ever, has received any definite idea in the personal care of this mouth.

The frequency of treatment is one of its greatest virtues for thereby no deposit can remain for any length of time, and the tissue is repeatedly stimulated and given opportunity to recover from hurtful influences.

Again to quote Dr. Smith: "The prophylaxis treatment as yet but very imperfectly understood by its friends, has been charged as merely a form of tooth-cleaning; far more than this, it is a manipulative process that positively relieves the teeth from a virulent infection and introduces a stimulation most beneficial to their internal and external life. If a tooth-cleaning process, it is one of profound significance."

### SPECIFIC RESULTS OF ORAL PROPHYLAXIS.

As to specific results obtained in the carrying out of oral prophylaxis, we find that it *does prevent* decay of the teeth, gingivities and pyorrhea alveolaris, and will cure most cases of pyorrhea if not advanced too far.

It will reduce inflammation in the soft tissues of the mouth.

It lessens sensation in teeth in all localities, especially at the cervix, and checks erosion.

A change in the appearance of the teeth for the better is produced, giving them a brilliancy and more life-like appearance than is seen in untreated teeth.

Much disturbance of the digestive tract is remarkedly lessened, if not entirely cured by the adoption of prophylaxis methods.

The breath loses its disagreeable odor upon the reduction of putrefactive processes in the mouth, and salivary secretions become normal.

Thorough mastication creates a desire for the simpler foods, and prophylaxis places and keeps the mouth in such a comfortable condition that food taken into it is masticated from the pleasure it gives; whereas, food taken into an unhealthy mouth is hurriedly passed, almost unchanged, into the stomach, except to be somewhat contaminated by infectious material.

Resistance to infectious diseases is increased by lessening the number of germs in the mouth and raising the vitality of its tissues.

One great result obtained is the interesting of the patient in the care he gives his teeth himself and educating him to a keen sense of any uncleanliness in his mouth. He cannot know the difference between a healthy mouth and an unhealthy one until he has been under treatment for a time.

It is difficult for the dentist who has not followed prophylaxis methods to realize the far reaching results obtained by them, but a year's conscientious attention to this work will fully convince him that there is no service in dentistry of greater importance to his patient and none more gratifying to himself.

